# Effects of Hook Maneuver on Oxygen Saturation Recovery After −40 m Apnea Dive—A Randomized Crossover Trial

**DOI:** 10.3390/sports13010024

**Published:** 2025-01-15

**Authors:** Francisco DeAsís-Fernández, Álvaro Reina-Varona, Evangelos Papotsidakis, Juan Lafuente, José Fierro-Marrero

**Affiliations:** 1 Department of Physical Therapy, Occupational Therapy, Rehabilitation and Physical Medicine, Rey Juan Carlos University, 28922 Madrid, Spain; fasis.fernandez@urjc.es; 2 Motion in Brains Research Group, Centro Superior de Estudios Universitarios La Salle, Universidad Autónoma de Madrid, 28023 Madrid, Spain; jose.fierromarrero@yahoo.com; 3 Departamento de Oxigenoterápia Hiperbárica, CRIS-UTH, Hospital Moisses Brogges, 08970 Barcelona, Spain; vagp17@gmail.com; 4 Centro Médico Mapfre, 08009 Barcelona, Spain; lafuenj100@gmail.com

**Keywords:** breath-hold dive, hypoxia, hypoxic syncope, lung squeeze, ultrasound, drowning

## Abstract

To reduce the risk of syncope, trained breath-hold divers (BHDs) use a specialized breathing technique after surfacing called “hook breathing” (HB). It consists of a full inspiration followed by a Valsalva-like maneuver and with subsequent exhalation performed against resistance to generate continuous positive airway pressure during exhalation. This study analyzed the influence of HB on oxygen saturation recovery after a −40 m depth apnea dive in trained BHDs. Thirteen BHDs performed two dives to −40 m at different days, one followed by HB after a dive and the other using usual breathing (UB). To detect signs of lung edema, ultrasound B-line measurements were conducted before, 10 min after the dive, and within 1 h after the dive. To detect oxygen saturation recovery, pulse oximetry was recorded before and immediately after surfacing. Both groups exhibited significant increases in SpO_2_ over time (UB: F (2.25, 24.7) = 22.1, *p* < 0.001, ηg2 = 0.612; HB: F (2.11, 23.2) = 29.0, *p* < 0.001, ηg2 = 0.688). Significant differences in SpO_2_ were observed between the HB and UB groups at 30–45 s post-apnea, with higher SpO_2_ values in the HB group; between 1.64 and 5.08% of SpO_2_ in favor of the HB intervention. Four participants showed ultrasound B-lines within ten minutes post-dive. After a 40 m apnea dive, the results revealed significant SpO_2_ recovery from 30 s to 45 s, with the HB recovering more rapidly. No differences were found at earlier (10–25 s) or later time points (50–60 s).

## 1. Introduction

Breath-hold divers (BHDs) face a great risk of drowning immediately after surfacing from a dive, when oxygen saturation (SpO_2_) is very low [1]. At this moment, the sympathetic nervous system is reactivated, which accelerates metabolism in an attempt to recover from the effort of the previous apnea [2]. This heightened metabolism requires efficient breathing to prevent the remaining oxygen from being quickly consumed. While safety divers are present in competitive events, this is rarely the case in amateur freediving practices. According to the World Health Organization, drowning is the third leading cause of death from unintentional injury worldwide, with an estimated 236,000 drowning fatalities each year. Despite the challenges in accurately collecting data, this makes drowning a significant public health issue globally [3].

Most drowning incidents in amateur BHDs are caused by apnea-related hypoxemia, which can lead to a loss of motor control or syncope [4]. In trained BHDs, the occurrence of lung edema can exacerbate hypoxemia more quickly. When these divers descend beyond their residual volume (typically 25–40 m), lung volume can no longer contract due to thoracic stiffness. Simultaneously, blood flow redistributes from the periphery to the central regions, a phenomenon known as blood shift. This shift creates lower pressure in the lungs compared to the surrounding capillaries. As the pressure in the capillaries increases, blood extravasates into the lungs [5]. Even mild edema can significantly impair gas exchange, further raising the risk of syncope [6].

Pulse oximetry has been used as a simple and effective method to detect possible edema after diving [6,7]. In recent years, the use of lung ultrasound has been implemented as a more reliable method to detect post-dive pulmonary edema. With lung ultrasound, B-lines can be counted on images of different lung sections to quantify the presence of lung edema [8].

To prevent syncope induced by hypoxemia, trained BHDs use a breathing technique after surfacing called “hook breathing” (HB) [9]. It consists of a full inspiration interrupted at the beginning of exhalation with a Valsalva-like maneuver and with subsequent exhalation performed against resistance to generate continuous positive airway pressure during exhalation [6]. This breathing maneuver is observed in air force pilots to prevent loss of consciousness at high G-forces due to the drop in blood pressure during acceleration [10]. In BHDs, continuous positive airway pressure during expiration could open the atelectasis zones of the lung and force out edema-associated fluid [11]; also, the increase in the partial pressure of O_2_ achieved in the alveoli with HB would probably increase gas exchange [12]. A previous study showed how HB was effective in SpO_2_ recovery, after −30 m apnea, in BHDs with a possible (estimated by pulse oximetry) mild pulmonary edema, while in asymptomatic BHDs, the effect was insignificant [6].

The hook maneuver, a novel technique that has been traditionally used to prevent loss of consciousness in air force pilots [10], was described by Schagatay in 2012 [13] and has been studied previously as a breathing maneuver to reduce the recovery time of oxygen saturation after a −30 m depth apnea dive in trained BHDs [6]. This study aimed to analyze the influence of HB on SpO_2_ recovery after a −40 m deep dive compared to usual breathing (UB). We hypothesize a faster SpO_2_ recovery associated with HB.

## 2. Materials and Methods

A randomized crossover trial was conducted with a 1:1 allocation ratio assigning subjects to either perform: (1) HB followed by UB, or (2) UB followed by HB. Randomization was conducted prior to the assessment and initiation of the intervention using GraphPad 8 software (GraphPad Software Inc., La Jolla, CA, USA). The assessor was blinded as he did not know which participants were assigned. The washout period was 72 h. The trial was approved by the local Ethics Committee for Human Research (CSEULS-PI-215/2018) and was conducted in accordance with the ethical standards of the Helsinki Declaration. Protocol registration was performed in Clinical Trials Gov (NCT04366414).

### 2.1. Participants

A convenience non-probabilistic sampling method was employed, and the following inclusion criteria were imposed: (1) age between 18 and 65 years; (2) baseline ≥ 95% SpO_2_. The following exclusion criteria were applied: (1) cardiovascular diseases; (2) respiratory diseases; (3) lung squeeze event in the week prior to measurement; (4) current pharmacological treatment; (5) recreational drug use; (6) self-harming behaviors; (7) pregnancy or possibility of being pregnant; (8) splenectomy; and (9) vigorous physical activity within 24 h before this study.

The participants attended the intervention site (Apnea Canarias Center, Tenerife, Spain) on two separate days. Once there, the intervention protocol was explained. Subsequently, time was given for the reading and signing of informed consent, followed by the assessment.

### 2.2. Intervention

Each BHD performed two free immersion (FIM) dives to a −40 m depth separated 72 h apart, one followed by recovery breathing using HB and the other using UB. The starting order of the breathing maneuvers (HB or UB) was determined by randomization. All BHDs used their usual breathing preparation at the surface before the dives. The diver was asked to start the dive when feeling ready. The BHDs then used their arms to pull themselves down along a vertical line and up again, followed by recovery breathing using either HB or UB. The use of a personal belt and neck weight was allowed during the dive.

All BHDs, already familiar with the HB protocol, received the following information: “take deep inhale, hold your breath and start to exhale with your glottis closed; then, open it, and continue to exhale with mouth resistance. When you finish, inhale and repeat protocol for one minute”.

### 2.3. Outcome Measures

The participants’ sociodemographic and other characterization variables were gathered, such as (1) age; (2) sex; (3) body mass index (BMI); (4) resting heart rate (HR); (5) resting SpO_2_; (6) years of apnea experience (in years, number of competitions and personal best in FIM); (7) history of apnea dive competitions (number); (8) personal best in FIM; and (9) percentage of the depth reached (40 m) related to the personal best in FIM.

Baseline ultrasound B-lines were recorded before the competition, with participants not having conducted diving activity 24 h before the assessment. Lung ultrasound was collected in the seated position by displaying mode B, using 2D ultrasonic imaging, with a convex transducer 3.5 MHz (Chison ECO 5 Portable Ultrasound Scanner, Wuxi, China). Bilateral imaging of the hemithorax from the second to fourth intercostal spaces was performed, culminating in 6 assessment zones.

A B-line was detected as an echogenic comet-shaped signal spreading from the pleural line to the further border of the screen. The same medical researcher (E-P) performed all ultrasound measurements; then, B-lines were analyzed by another medical researcher specialist on pulmonary ultrasound (J-L) to provide a total B-line score [14]. Ultrasound B-lines are an index of extravascular lung fluid, and the sensitivity and reliability of this method have been contrasted in different studies compared with radiographic imaging [15,16]. After analysis, the total number of B-lines was recorded to detect possible signs of pulmonary edema.

After surfacing, the SpO_2_ and HR recordings started as soon as possible. Pulse oximetry (NoninPalmSAT^®^ 2500 series, Nonin Medical Inc, Tilburg, The Netherlands) was recorded every 5 s during 1 min, while BHDs performed either the HB or UB protocol. During the measurement, a signal on the pulse oximeter indicated with a green light (good perfusion) or a red light (bad perfusion) if readings were reliable. Only data with good finger perfusion indicated were included. In further analysis, the seconds the participant took to reach normoxia were counted (95% SpO_2_; [17]). If the diver did not reach this value during recovery, 60 s were counted.

At the end of the pulse oximetry recording, participants went by boat (within 10 min post-dive) to Radazul Port in Tenerife, where a new lung ultrasound (same protocol to baseline) was performed to detect possible signs of pulmonary edema. The data were stored and sent to the medical assessor, who was blinded to the interventions; however, if the participant showed obvious signs of edema (hemoptysis, chest tightness, dyspnea, hypoxemia), they would immediately be excluded from the study and transferred to a medical center for further examination. One hour after the end of the dive, the participants were called back to evaluate, again, their condition by pulse oximetry and lung ultrasound. This procedure was conducted in accordance and approved by the Ethics Committee.

### 2.4. Statistical Analysis

All statistical analyses were performed in R (version [4.4.1]) within the RStudio environment (RStudio, PBC, Boston, MA, USA) version 2023.06.0 + 421. A range of R packages were utilized to ensure robust data manipulation, visualization, and statistical analysis, including tidyverse for data processing, rstatix for repeated-measures ANOVA and post hoc tests, and ggplot2 for visualization.

Descriptive statistics summarized the sociodemographic, characterization, and outcome variables, presenting mean ± SD, median, quartiles, minimum and maximum values, and absolute frequencies and percentages for categorical variables. Data visualizations, such as boxplots, illustrated the distributions of SpO_2_ values for each group over time.

A repeated-measures ANOVA was conducted to examine the effects of group (breathing technique: HB vs. UB) and time (11 post-apnea intervals) on SpO_2_. These variables were included as two within-subject factors. The model accounted for within-subject variability using participant ID as the repeated factor. Assumption of normality was assessed with Q-Q plots for each group × time combination, and Mauchly’s test assessed the assumption of sphericity. Greenhouse–Geisser corrections were applied where sphericity was violated to adjust the degrees of freedom. One participant was eliminated from the analysis due to deviation from normality. After examining this subject, he was significantly older than the rest of the participants and he had developed severe pulmonary edema in both conditions, which may have produced a delayed SpO_2_ recovery across time.

Differences in SpO_2_ between the HB and UB conditions accounted for the main effect of group. Time-dependent changes in SpO_2_ irrespective of breathing technique accounted for the main effect of time. Finally, differences in SpO_2_ between breathing techniques across time points accounted for the interaction effect between group and time. Significant effects, which were set at *p* < 0.05, were followed-up with post hoc analyses to explore specific differences.

Post hoc comparisons were performed with Bonferroni corrections for between groups at each time point and within groups across time points. Pairwise comparisons tested SpO_2_ differences using paired *t*-tests. Significant differences were reported with mean differences (MDs) and 95% confidence intervals (95% CIs).

## 3. Results

### 3.1. Descriptive Statistics

The sample consisted of 13 professional breath-hold divers with a mean age of 42 ± 9 years and a mean BMI of 24 ± 1.4 kg/m^2^. All participants were male with an average apnea experience of 7.2 ± 2.9 years and had attended 11 ± 14 competitions. Baseline SpO_2_ (%) was 97 ± 0.69 for the UB group and 97± 0.69 for the HB group, and baseline HR was 69 ± 16 bpm for the UB group and 70 ± 15 bpm for the HB group. All descriptive information is presented in Table 1 and Table 2.

**Figure 1:**
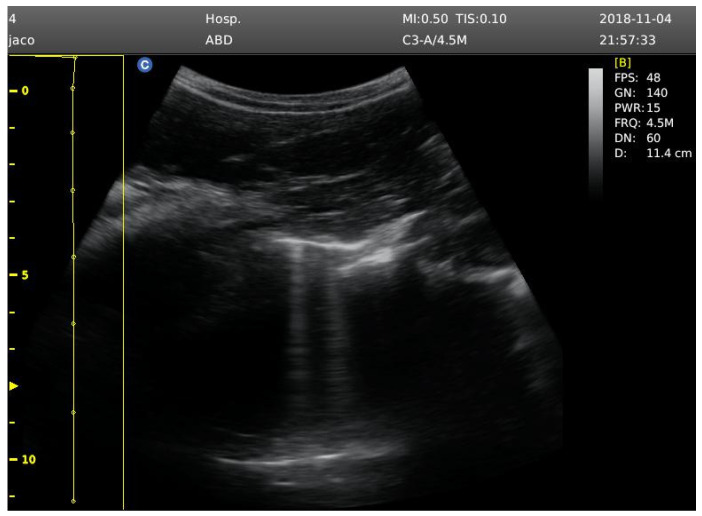
Signs of ultrasound B-lines. Image analyzed at 12 cm depth.

Four participants showed signs of mild pulmonary edema, after diving, coinciding in both protocols (Supplementary Materials Table S1). The ultrasound B-lines count was recorded at baseline, 10 min post-immersion, and at 1 h post-immersion (Figure 1).

### 3.2. Oxygen Saturation Recovery

The repeated-measures ANOVA revealed significant main effects for group [F (1, 11) = 5.254; *p* = 0.043, ηg2 = 0.014] and time [F (2.08, 22.87) = 32.171, *p* < 0.001, ηg2 = 0.649], as well as a significant group × time interaction [F (3.30, 36.26) = 3.497, *p* = 0.022, ηg2 = 0.054].

### 3.3. Oxygen Saturation Recovery—Post Hoc Analysis

#### 3.3.1. Within-Group Time Comparisons

Both groups exhibited significant increases in SpO_2_ over time [UB: F (2.25, 24.7) = 22.1, *p* < 0.001, ηg2 = 0.612; HB: F (2.11, 23.2) = 29.0, *p* < 0.001, ηg2 = 0.688]. Pairwise comparisons revealed significant SpO_2_ recovery from 10 s to 35 s, with the HB group recovering more rapidly to near-baseline levels.

#### 3.3.2. Between-Group Comparisons

**Figure 2:**
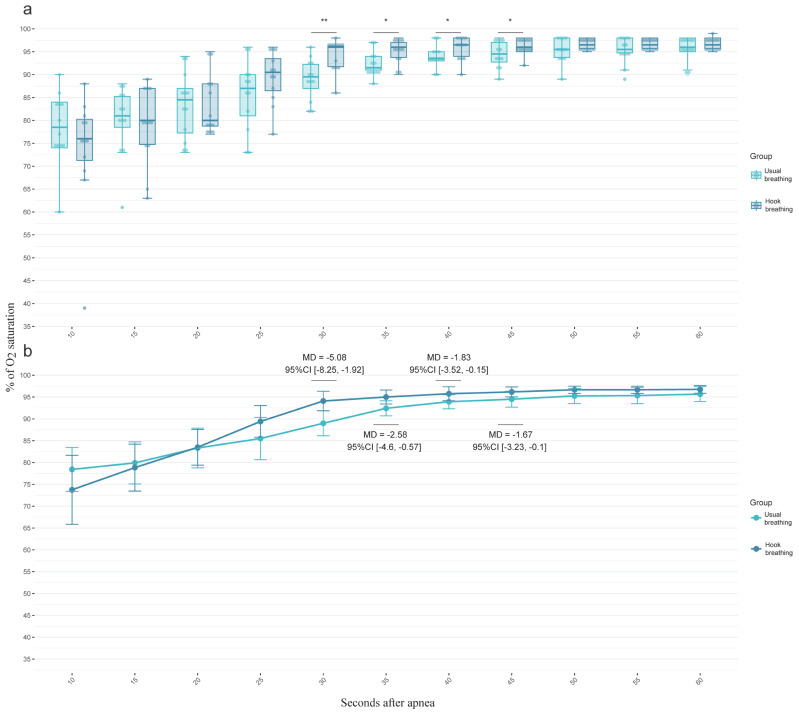
Percentage of oxygen saturation for both groups per each time, showing groups’ distributions (**a**) and means (**b**). Differences are shown in mean differences (MD) and 95% confidence intervals (95%CI). * <0.05; ** <0.01.

Significant differences in SpO_2_ were observed between HB and UB at 30–45 s post-apnea, with higher SpO_2_ values in the HB group. Significant differences between groups included mean difference values ranging between 1.64 and 5.08% of SpO_2_ in favor of HB intervention. No significant differences were found at earlier (10–25 s) or later time points (50–60 s). Differences between groups are shown in Figure 2.

## 4. Discussion

This study aimed to analyze the influence of HB on the recovery of oxygen saturation after a 40 m deep dive. To check oxygen saturation recovery, each participant was monitored by pulse oximetry during the first minute after apnea. The results showed an improved oxygen recovery in both groups; however, between 30 and 45 s, HB reached faster oxygen recovery. No significant differences were found at earlier or later time points. One explanation might be reflected in Table 2, where it can be observed that in the HB protocol, participants surfaced with significantly lower saturations (5% lower on average) than in the UB protocol. This could explain why there are no differences at the beginning of the recovery, as the hemoglobin dissociation curve does not have linear but exponential behavior [18]. Also, the time elapsed during surface breathing between alveolar capillaries and peripheral arteries (finger pulse oximetry) could support why SpO_2_ changes after 30 s, i.e., that the first 20 s does mostly reflect the peripheral vasoconstriction of the dive [19,20]. During the later time points, after 50 s, there is a clearer hypothesis, which is that when normoxia is reached in both groups, a physiological ceiling effect is produced.

The recovery of oxygen saturation after a deep apnea dive is primarily driven by the body’s mechanisms for re-establishing normal oxygen levels following hypoxia and hypercapnia [19]. During the dive, oxygen is depleted from the bloodstream as the body relies on stored oxygen in the lungs and tissues. Upon surfacing, oxygen begins to be replenished through the lungs, with hemoglobin binding to the incoming oxygen and restoring arterial oxygen levels. The rate at which SpO_2_ recovers might be influenced by factors such as depth, apnea time, the aerobic capacity, or the use of specific breathing techniques [6], which may assist in oxygen uptake.

Participants performed a deep dive to 40 m. At this depth, the residual volume is normally reached [21]. In this condition, because of the blood shift, small extravasations of liquid from the surrounding blood vessels into the alveolus might occur, resulting in a predictably mild pulmonary edema [20]. The initial hypothesis suggested a faster oxygen saturation recovery associated with HB, pronounced in divers suffering with mild lung edema. The proposed mechanism is that the increased pulmonary pressure with HB will counteract the pulmonary edema developed and facilitate oxygen uptake in divers prone to squeeze. This was supported by a previous study, where the results suggest that HB is efficient for increasing the oxygen saturation recovery rate in individuals prone to squeeze; however, signs of edema were estimated by pulse oximetry [6]. In the present study, after a pulmonary ultrasound test, the same four participants (30% of total) showed ultrasound B-lines within ten minutes post-dive. These signs of mild pulmonary edema were observed in both protocols (HB and UB; see Supplementary Materials Figure S1). As the sample was so small, it was not feasible to establish a subgroup analysis of those participants with signs of mild edema, and the descriptive data are merely reflected in the results.

Ultrasound B-lines were performed again one hour after to detect possible areas of atelectasis caused by pulmonary edema [22]. This is important, since one of the effects to be studied was the effect of HB on the recovery of fluid accumulated during extravasation [8]. In addition to lung ultrasound, oxygen saturation recovery was observed by pulse oximetry after one hour to see if the release of atelectasis zones produced improvements in alveolar diffusion and therefore in oxygen saturation. The results revealed that normoxia was reached after one hour in both groups (1% SpO_2_ higher in HB). Regarding ultrasound B-lines, they also showed a significant reduction in both groups, but only asymptomatic B-Lines were reached by HB after one hour in BHDs with mild lung edema. Possible effects of HB, in addition to opening up atelectatic areas of the lung and forcing out any fluid, could possibly be due to the slightly increased partial pressure of O2 achieved with HB [10]. Other possibly contributing effects of HB could be related to changes in pulmonary ventilation/perfusion, venous return, and blood pressure, which could be further studied in the laboratory [23].

This study has certain limitations that are important to highlight. Firstly, the experimental diving depths were submaximal for most of the studied divers and may not have been straining for the pulmonary system, but it is likely that a greater portion of the divers would respond with a mild lung edema in maximal competition dives well beyond their individual residual volume. Also, the heterogeneity of the stimulus at 40 m deep (with difference between 32% and 100% from their personal best in FIM) might produce heterogeneous responses.

Although ultrasound was performed as soon as possible without compromising the dive safety protocol, ten minutes passed from the dive to the pulmonary ultrasound test. This might reduce ultrasound B-lines due to the recovery time as indirect measurement pulse oximetry was performed immediately after surfacing to detect signs of hypoxemia, which could indicate mild pulmonary edema if normoxia was not restored within the first minute after the dive. When participants surfaced, the safety diver took their hands close to the boat where one of the researchers was, and after drying the finger, it was placed in a pulse oximeter, which took a few seconds to detect the data. This explains why there is no oxygen saturation data for the first 10 s after the dive.

While this sample size can provide valuable information, it is important to consider that larger sample sizes are often recommended for variables to have a distribution closer to normality and to allow for better generalization of the results. In future studies, it is suggested to consider a greater sample size to increase the robustness of the statistical analyses and strengthen the external validity of the findings [24].

The results suggest that HB might be efficient in speeding up recovery in individuals with and without mild lung edema, but a larger sample size is suggested to detect a reliable effect size of such an intervention. With a large heterogeneity of post-dive breathing protocols, HB is probably the recovery protocol that has shown the greatest size effect based on plausible scientific hypotheses.

Future studies should focus on investigating the potential protective effects of HB on lung edema, as well as comparing HB to other recovery techniques, to offer valuable insights into optimizing post-dive recovery. Additionally, studies should delve into the physiological mechanisms behind faster SpO_2_ recovery with HB. Controlling factors like environmental conditions, hydration, and training levels, and including divers with varying experience, could further refine our understanding of HB’s effectiveness.

## 5. Conclusions

After a 40 m apnea dive, the results revealed significant SpO_2_ recovery from 30 s to 45 s in individuals with and without mild lung edema, with the HB group recovering more rapidly to near-baseline levels. No differences were found at earlier (10–25 s) or later time points (50–60 s).

## Figures and Tables

**Table 1:** Sociodemographic and baseline data of participants. % PB: percentage of personal best in free immersion; BMI: body mass index; PB FIM: personal best in free immersion.

Variable	N (%)	Mean ± SD	Min	Q1	Median	Q3	Max
Age (years)	13	42 ± 9	32	36	42	45	65
Sex							
Male	13 (100%)						
Height (cm)	13	179 ± 9.4	162	174	180	183	198
Body mass (kg)	13	79 ± 11	60	72	78	85	102
BMI (kg/m^2^)	13	24 ± 1.4	21	23	25	26	26
Apnea experience (years)	13	7.2 ± 2.9	4	5	6	8	13
PB FIM	13	57 ± 23	40	43	46	60	124
% PB FIM	13	78 ± 21	32	67	87	93	100

Note. BMI, body mass index; PB FIM, personal best in free immersion; %PB FIM, percentage of personal best in free immersion.

**Table 2:** Oxygen saturation descriptive data in baseline and during sixty seconds recovery post-dive. SpO_2_: oxygen saturation by pulse oximetry.

Variable	N	Mean ± SD		Min	Q1	Median	Q3	Max
Baseline values								
SpO_2_ (%)								
UB	13	97 ± 0.75		96	96	97	97	98
HB	13	97 ± 0.69		96	97	97	98	98
**Post-immersion recovery**								
SpO_2_ (%)—10 s								
UB	13	78 ± 8.2		60	74	77	84	90
HB	13	73 ± 12		39	70	76	80	88
SpO_2_ (%)—15 s								
UB	13	79 ± 8.2		61	74	80	85	88
HB	13	78 ± 8.6		63	74	80	87	89
SpO_2_ (%)—20 s								
UB	13	82 ± 8.4		66	75	83	86	94
HB	13	82 ± 7.7		67	78	79	88	95
SpO_2_ (%)—25 s								
UB	13	84 ± 9.3		65	78	86	90	96
HB	13	88 ± 8.7		65	85	90	93	96
SpO_2_ (%)—30 s								
UB	13	87 ± 7.9		65	84	89	92	96
HB	13	92 ± 9		64	91	96	96	98
SpO_2_ (%)—35 s								
UB	13	90 ± 8		65	91	91	94	97
HB	13	93 ± 7.9		68	93	96	97	98
SpO_2_ (%)—40 s								
UB	13	92 ± 8.4		65	93	93	95	98
HB	13	94 ± 6.8		73	94	96	98	98
SpO_2_ (%)—45 s								
UB	13	92 ± 8.9		64	92	94	97	98
HB	13	95 ± 5.6		77	95	96	98	98
SpO_2_ (%)—50 s								
UB	13	93 ± 9.3		63	93	95	98	98
HB	13	95 ± 4.8		80	95	96	98	98
SpO_2_ (%)—55 s								
UB	13	93 ± 8.9		65	94	95	98	98
HB	13	96 ± 4.3		82	95	96	98	98
SpO_2_ (%)—60 s								
UB	13	93 ± 8.9		65	95	96	98	98
HB	13	96 ± 2.8		88	95	96	98	99

Note. HB, HB breathing; UB, usual breathing; SpO_2_, oxygen saturation by pulse oximeter.

## Data Availability

The data that support the findings of this study are available upon request to the corresponding author.

## Supplementary Materials

The following supporting information can be downloaded at: https://www.mdpi.com/article/10.3390/sports13010024/s1, Table S1: Count of ultrasound B-lines. Figure S1: Q-Q plot ANOVA.
