# Supplementary material for: Effects of Hook Maneuver on Oxygen Saturation Recovery After −40 m Apnea Dive—A Randomized Crossover Trial
Source: Sports (Basel). 2025 Jan 15;13(1):24. doi: 10.3390/sports13010024 (PMC11768466; doi:10.3390/sports13010024)
Supplement: Supplementary file 1 [file sports-13-00024-s001.zip › Supplementary material Table and Figure.pdf]

Supplementary Data S1. Count of ultrasound B-lines

ID	Nº B-lines 10 min post dive		Nº B-lines 1 hour post dive	
	UB	HB	UB	HB
**1**	0	0	0	0
**2**	3	0	1	0
**3**	0	0	0	0
**4**	27	15	11	0
**5**	0	0	0	0
**6**	0	0	0	0
**7**	11	1	8	0
**8**	0	0	0	0
**9**	0	0	0	0
**10**	10	7	8	0
**11**	0	0	0	0
**12**	0	0	0	0
**13**	0	0	0	0

HB, Hook Breathing; UB, Usual Breathing.

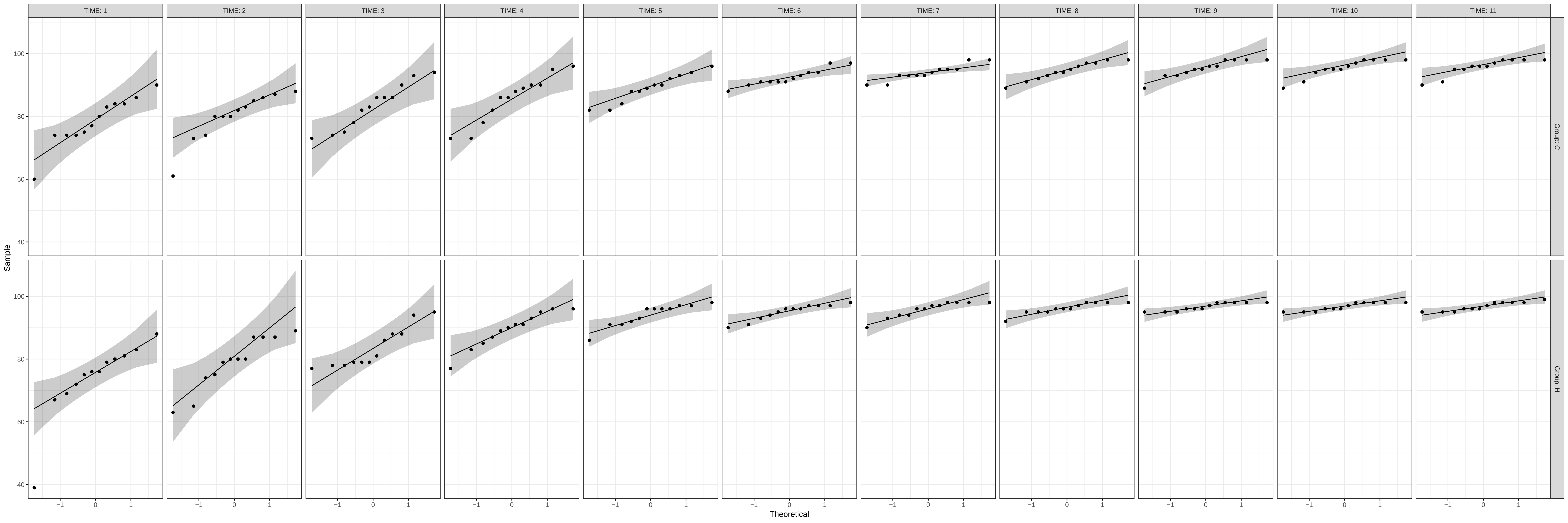
TIME: 1
TIME: 2
TIME: 3
TIME: 4
TIME: 5
TIME: 6
TIME: 7
TIME: 8
TIME: 9
TIME: 10
TIME: 11
Group: C
Group: H
Sample
Theoretical
